# Adipogenic miRNA and meta-signature miRNAs involved in human adipocyte differentiation and obesity

**DOI:** 10.18632/oncotarget.8518

**Published:** 2016-03-31

**Authors:** Chunmei Shi, Fangyan Huang, Xiaohong Gu, Min Zhang, Juan Wen, Xing Wang, Lianghui You, Xianwei Cui, Chenbo Ji, Xirong Guo

**Affiliations:** ^1^ Department of Children Health Care, Nanjing Maternity and Child Health Care Hospital Affiliated to Nanjing Medical University, Nanjing, China; ^2^ Institute of Pediatrics, Nanjing Medical University, Nanjing, China

**Keywords:** miRNA, adipocyte, adipogenesis, meta-analysis, obesity

## Abstract

MicroRNAs (miRNAs) have been identified as a new class of regulatory molecules that influence many biological functions, including metabolism, adipocyte differentiation. To determine the role of adipogenic miRNAs in the adipocyte differentiation process, we used microarray technology to monitor miRNA levels in human adipose-derived mesenchymal stem cells (hMSCs-Ad), human stromal vascular cells (SVCs) and differentiated adipocytes. 79 miRNAs were found to be differentially expressed, most of which are located in obesity related chromosomal regions but have not been previously linked to adipocyte differentiation process. A systematic search was made for relevant studies in academic data bases, involving the Gene Expression Omnibus (GEO) ArrayExpress, Pubmed and Embase database. Eight studies on human adipocyte differentiation or obesity were included in the final analysis. After combining our microarray data with meta-analysis of published microarray data, we detected 42 differently expressed miRNAs (meta-signature miRNAs) in mature adipocytes compared to SVCs or hMSCs-Ad. Our study shows meta-signature miRNAs specific for adipogenesis, several of which are correlated with key gene targets demonstrating functional relationships to pathways in BMP signaling pathway, Cell differentiation, Wnt signaling, insulin receptor signaling pathway, MAPK signaling, Cell cycle and lipid metabolic process. Our study shows that the first evidence of hsa-let-7 family, hsa-miR-15a-5p, hsa-miR-27a-3p, hsa-miR-106b-5p, hsa-miR-148a-3p and hsa-miR-26b-5p got a great weight in adipogenesis. We concluded that meta-signature miRNAs involved in adipocyte differentiation and provided pathophysiological roles and novel insight into obesity and its related metabolic diseases.

## INTRODUCTION

Obesity has become a major worldwide health problem associated with metabolic and cardiovascular disorders. Obesity is a progressive and multifactorial pathology characterized by excessive expansion of white adipose tissue, which due to an increase in adipocyte hyperplasia and hypertrophy [1]. Since obesity-related hyperplasia and hypertrophy are often associated with various metabolic disorders, proper understanding of the molecular events regulating adipogenesis and lipid metabolism can provide valuable information for exploiting comprehensive and effective therapeutic strategies against obesity.

MicroRNAs (miRNAs), small non-coding RNAs of 18–25 nucleotides in length, which were seen to control gene expression in virtually obesity or other metabolism syndrome, were abundantly investigated relating to proliferation and differentiation of adipogenesis. Indeed, the role of some miRNAs has been described in obesity. For example, miR-103, miR-107, and miR-143 regulate liver insulin sensitivity in diet-induced obesity through different mechanisms [2, 3]. The involvement of miRNAs in obesity pathogenesis is well established, as they can behave as pro-adipogenic or adipogenic suppressor genes depending on the cellular function of their targets [4].

Emerging evidence shows that miRNAs may directly or indirectly modulate adipocyte differentiation. In our previous study, we found that miR-148a, as a biomarker of obesity, promoted adipogenesis by inhibiting Wnt1 [5]. miR-146b as a positive regulator of accelerated adipocyte differentiation through modulation of KLF7 [6]. Recent findings [7] have suggested that miR-26b inhibits adipogenic differentiation and overexpression of miR-1908 inhibited adipogenic differentiation [8]. However, to date, there is no meta-analysis to interpret the biology of miRNA and its contribution to obesity development which may provide early promising therapeutic targets and effective control of obesity.

In the present study, using a miRNA array strategy, we identified 79 differentially expressed miRNAs, most of which located in obesity related chromosomal regions but have not been previously linked to adipocyte differentiation process. Additionally, to overcome the limitations in current researches, we performed meta-analysis applying the robust rank aggregation method [9], followed by pathway analysis, to identify miRNA regulation in adipogenesis and the pathways that key miRNAs may impact. Identification of miRNA meta-signature and involved pathways would provide the potential target and novel insight into obesity and its related metabolic diseases.

## RESULTS

### miRNA microarray results

**Figure 1:**
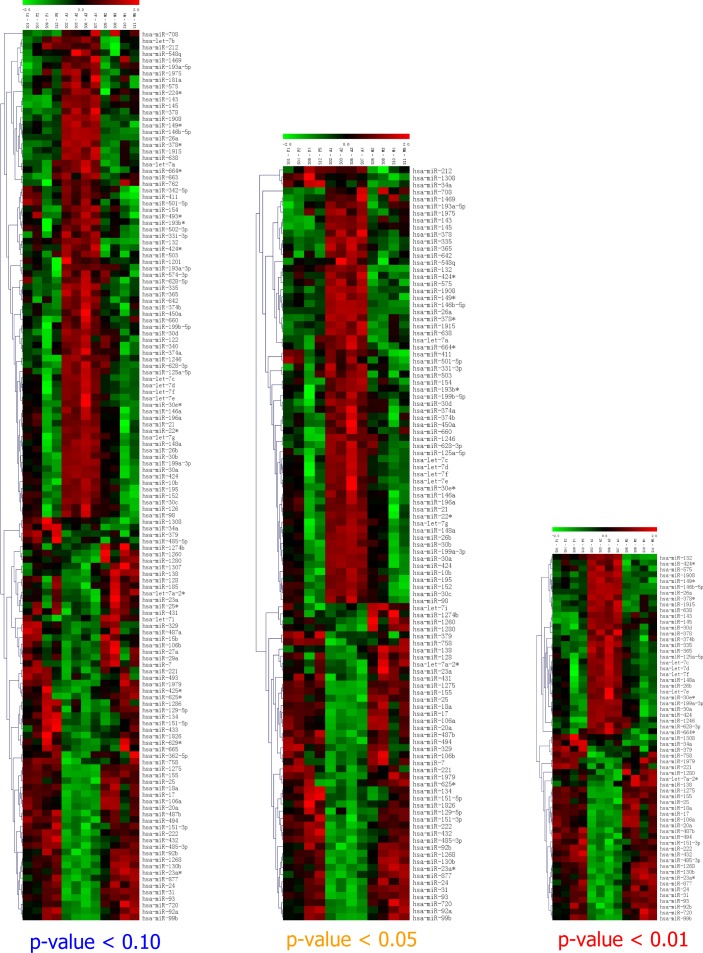
Heatmap of results of microRNA microarray analysis Up-regulated microRNAs are shown in red, and downregulated microRNAs are shown in green. The fold-changes (log2 transformed) of miRNA expression in differentiated *versus* SVCs or undifferentiated hMSCs-A. Each group (A1-A4, PA1-PA4 and M1-M4) is pooled from three samples. A: mature adipocyte; SVCs (PA: preadipocyte): human stromal vascular cells; M: undifferentiated hMSCs-Ad.

miRNA microarray analysis was performed to identify differentially expressed miRNAs in mature adipocytes and then compared to SVCs and hMSCs-Ad. A larger number of microRNAs (79 miRNAs, 45 up-regulated miRNAs and 34 down-regulated miRNAs) were significantly up- or down-regulated in mature adipocytes compared to SVCs and hMSCs-Ad (Figure 1, Supplementary Table 1). Our data showed that differentially expressed miRNAs were overlapping either in mature adipocytes versus SVCs or in mature adipocytes versus hMSCs-Ad (Supplementary Table 1, *P*< 0.05). Between mature adipocytes and SVCs/hMSCs-Ad, miR-146b-5p and miR-335 were found to be differentially expressed with a fold change >10 (Supplementary Table 1). Whereas, miR-424 was differentially expressed with a fold change of more than 10 in mature adipocytes when compared to hMSCs-Ad, and the expression level was about 9.2-fold when compared to SVCs. Of all differentially expressed miRNAs, miR-1275, miR-155 and miR-1268 were down-regulated in mature adipocytes over 10-fold when compared with SVCs and hMSCs-Ad. We identified that miR-148a, miR-26b, miR-132, miR-365 and miR-1908 were highly expressed in mature adipocytes with over 5-fold compared to SVCs/hMSCs-Ad. Additionally, miR-93 and miR-720 were lowly expressed with 5-fold in mature adipocytes compared to SVCs/hMSCs-Ad. Among these differentially expressed miRNAs, our group did further research for the adipogenic miRNAs *in vivo* and *in vitro*, including miR-148a [5], miR-26b [10], miR-146b [6] and miR-1275.

### PCR validation of significant differentially expressed miRNAs expression

**Figure 2:**
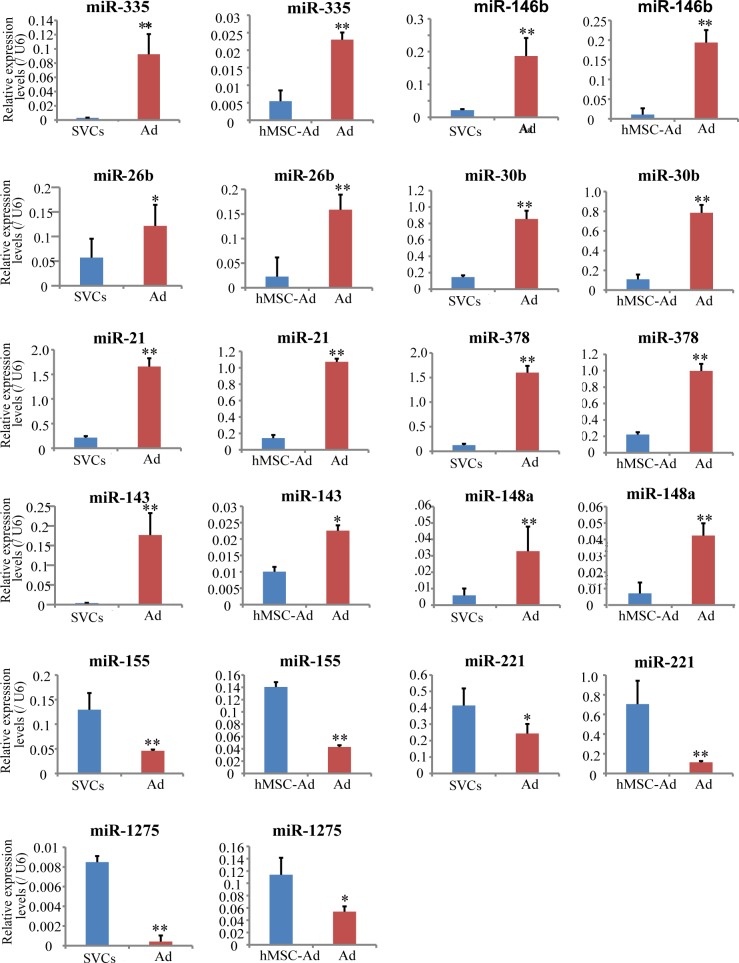
mRNAs expression in mature adipocytes *versus* SVCs or hMSCs-Ad.SVCs or hMSCs-Ad was grown to confluence and adipogenic differentiation was initiated as described in Research Design and Methods Expression of miRNAs during adipocyte differentiation was quantitated by TaqMan miR-based qRT-PCR. The bar is the mean ± SD of four independent experiments that were analyzed using Independent T-Test vs. undifferentiated SVCs or hMSCs-Ad. SVCs: human stromal vascular cells; hMSCs-Ad: undifferentiated hMSC-Ad; Ad: mature adipocyte.

To confirm the microarray hybridization results, qRT-PCR was performed on 8 up-regulated miRNAs (miR-335, miR-146b-5p, miR-26b, miR-30b, miR-21, miR-378, miR-143 and miR-148a) [10] and 3 down-regulated miRNA (miR-155,miR-221, and miR-1275), chosen on the basis of their levels of expression on the microarray and their biological significance. qRT-PCR was performed in triplicate on the diluted cDNA and the experiments were conducted twice on differentiated hMSCs-Ad/mature adipocytes and hMSCs-Ad/SVCs. The qRT-PCR results were closely mirrored with the microarray analysis showing *P*-value <0.05 (Figure 2).

### The location of differentially expressed miRNAs in obesity gene map

To elucidate the correlation pattern between miRNA and obesity, the chromosomal regions of differentially expressed miRNAs were performed using Genomic map view analysis. We compared the chromosomal regions of differentially expressed miRNAs with obesity gene map published in 2005 [11]. As shown in Table 1, there were about 65% (51/79) miRNAs located in obesity related chromosomal regions. Our results indicated that these differentially expressed miRNAs have a close relationship with obesity.

**Table 1:** Chromosome of Adipocytes specific miRNAs distribution compared with obese gene map

miRNA Name	Localization	Population	Phenotypes
hsa-miR-34a	1p36.22	>168 pairs	Skinfolds, uprailiac
hsa-miR-128-1	2q21.3	453 subjects, 99 families	Abdominal visceral fat
hsa-miR-26b	2q35	453 subjects, 99 families	Abdominal visceral fat
hsa-miR-128-2	3p22.3	377 pairs	Body fat (%)
hsa-miR-26a-1	3p22.2	580 families	BMI
hsa-miR-143	5q32	453 subjects, 99 families	Abdominal total fat
hsa-miR-145	5q32	453 subjects, 99 families	Abdominal total fat
hsa-miR-378	5q32	453 subjects, 99 families	Abdominal total fat
hsa-miR-25	7q22.1	261 subjects, 27 pedigrees, 1297 subjects, 260 families	High-density lipoprotein, in triglyceridesBMI
hsa-miR-93			
hsa-miR-106b			
hsa-miR-335	7q32.2	545 pairs	BMI
hsa-miR-29a	7q32.3	3027 subjects, 401 families, 317 sibships	BMI
hsa-miR-320a	8p21.3	994 subjects, 37 pedigrees	BMI
hsa-miR-1915	10p12.31	667 subjects, 244 families; 862 subjects, 170 families	Obesity (in whites and African Americans) Obesity (inAfrican Americans, in European Americans)
hsa-miR-605	10q21.1	672 subjects, 28 pedigrees; 862 subjects, 170 families	Leptin, Obesity
hsa-miR-146b	10q24.32	447 subjects, 109 pedigrees	BMI > 27
hsa-miR-130a	11q12.1	369 subjects, 89 families	Obesity (in white children and adolescents)
hsa-miR-125b-1	11q24.1	430 subjects, 27 sibpairs, 27 pedigrees;1526 pairs;1778 sibships;994 subjects, 37 pedigrees	BMI
hsa-let-7a-2			
hsa-miR-100			
hsa-miR-196a-2	12q13.13	729 subjects, 275 families	Waist-to-hip ratio
hsa-miR-26a-2	12q14.1	514 subjects, 99 families, 347 sibships	Fat intake
hsa-let-7i			
hsa-miR-16-1	13q14.2	3027 subjects, 401 families, 317 sibships	BMI
hsa-miR-17	13q31.3	1297 subjects, 260 families; 1297 subjects, 260 families; 1312 subjects, 696 families	BMI, paternal, Waist circumference, paternal; BMI; Factor central obesity
hsa-miR-18a			
hsa-miR-20a			
hsa-miR-19b-1			
hsa-miR-92a-1			
hsa-miR-1260	14q24.3	672 subjects, 28 pedigrees	Leptin
hsa-miR-1469	15q26.2	336 sibpairs, 609 relative pairs	Fat-free mass
hsa-miR-365-1	16p13.12	893 sibpairs	BMI (in whites)
hsa-miR-22	17p13.3	729 subjects, 275 families	Abdominal subcutaneous fat
hsa-miR-132	17p13.3		
hsa-miR-195	17p13.1	478 subjects, 10 families	asp levels
hsa-miR-423	17q11.2	470 subjects, 10 families	BMI
hsa-miR-365-2	17q11.2		
hsa-miR-152	17q21.32	521 subjects, 156 families	Abdominal subcutaneous fat
hsa-miR-196a-1	17q21.32		
hsa-miR-638	19p13.2	522 subjects, 99 families, 364 sibpairs	Skinfolds, sum of eight (in whites), Leptin (in whites), Body fat (%) (in whites)
hsa-miR-199a-1-5p	19p13.2		
hsa-miR-199a-1-3p	19p13.2		
hsa-miR-24-2	19p13.13	506 subjects, 115 pedigrees	Age adiposity rebound
hsa-miR-27a	19p13.13		
hsa-miR-23a	19p13.13		
hsa-miR-155	21q21.3	1510 subjects, 509 families	Factor central obesity
hsa-let-7a-3	22q13.31	>168 pairs	Body weight
hsa-let-7b	22q13.31		
hsa-miR-221	Xp11.3	1148 subjects, 133 families, 190 European-American families (940 members), 43 African-American families (208 members)	Waist-to-hip ratio (in European Americans and African Americans)
hsa-miR-222			

Note: The Human Obesity Gene Map: The 2005 Update

### Study selection and data extraction

**Figure 3:**
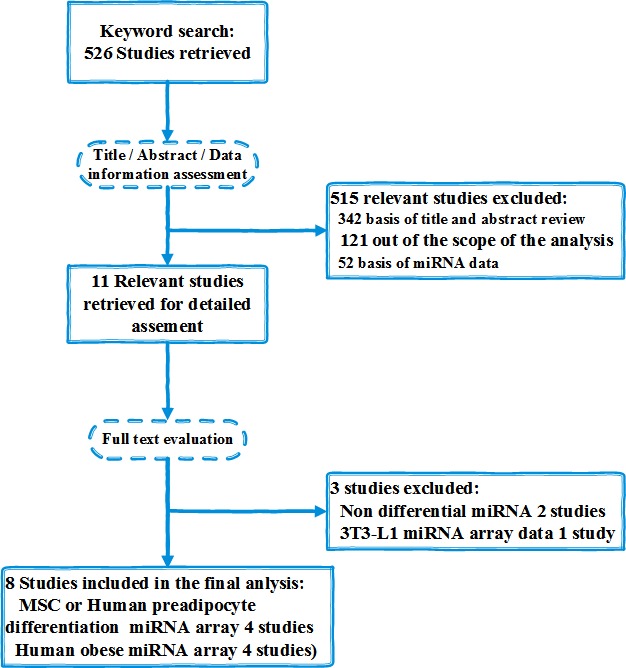
Flowchart of study selection

We retrieved publications by the term of “adipocyte OR fat cell OR adipogenesis OR MSC (mesenchymal stem cells) OR obesity OR adipocyte differentiation” AND “miRNA OR microRNA” AND human”. Database searches initially yielded a total of 526 publications and 8 studies met the inclusion criteria (Figure 3, Table 2). Our research was included in the final analysis because the lists of ranked miRNA were suitable for the inclusion criteria. Most of the studies were published between 2010 and 2014. 3 of the researches came from America and 5 of the researches came from Europe.

**Table 2:** Baseline characteristics of eligible studies

ID	link	panle	Group	Country	Fold change
[12]	https://www.gcbi.com.cn/gclib/html/pubmed/detail/20057369	GPL7162 CEINGE_Exiqon_Human miRNA Microarray [208002V8.1]	nondiabetic severely obese *Vs*. nonobese adults	Italy	No
[13]	https://www.gcbi.com.cn/gclib/html/pubmed/detail/25518011	GPL16384 [miRNA-3_0] Affymetrix Multispecies miRNA-3 Array	obese *Vs*. lean visceral adipocye exosomes	USA	Yes
[14]	http://www.ncbi.nlm.nih.gov/pubmed/22688341	[miRNA-1_0] Affymetrix miRNA Array	obese (*n* = 30) *Vs*. nonobese (*n* = 26)	Sweden	Yes
[15]	https://www.gcbi.com.cn/gclib/html/pubmed/detail/23493574	GPL8786 [miRNA-1_0] Affymetrix miRNA Array	adipocytes derived from PCOS patients vs. control	USA	Yes
[18]	https://www.gcbi.com.cn/gclib/html/pubmed/detail/20126310	GPL7731 Agilent-019118 Human miRNA Microarray 2.0 G4470B	obese *Vs*. normal	Spain	Yes
[16]	http://www.ncbi.nlm.nih.gov/pubmed/25356868	miRCURY LNA microRNA Array, 6th generation - hsa, mmu&rno [miRBase 17.0]	AD day 13 *Vs* AD day 0 adipocyte	Denmark	Yes
[17]	https://www.gcbi.com.cn/gclib/html/pubmed/detail/21756067	GPL11107 mirVana 1.3K v1.4 201205	adipocyte differentiation	Norway	no
[19]	https://www.gcbi.com.cn/gclib/html/pubmed/detail/20887899#	GPL8180 Illumina Mouse v1 MicroRNA expression beadchip	adipocyte differentiation	USA	YES

### Differently expressed miRNAs in meta-publications

As shown in Supplementary Table 2, Martinelli et al. [12] found 41 differentially expressed miRNAs in subcutaneous adipose tissue (SAT) from nondiabetic severely obese (n = 20) and non-obese adults (n = 8). Ferrante et al. [13] developed techniques to quantify and characterize exosomes shed by adipocytes from seven obese (age: 12–17.5 y, BMI: 33–50 kg/m^2^) and five lean (age: 11–19 y, BMI: 22–25 kg/m^2^) subjects and found 89 differentially expressed miRNAs between obese and lean visceral adipocyte. Arner et al. [14] identified 20 differentially expressed miRNAs in intact adipose tissue from obese (n = 30) and non-obese (n = 26) subjects. Chen et al. [15] detected 28 differentially expressed miRNAs in adipocytes of PCOS (polycystic ovary syndrome) patients and matched control subjects. Alajez et al. [16]used telomerase immortalized human bone marrow-derived stromal cell line (hMSC-TERT) to identify the differentially expressed miRNA; they identified 36 up-regulated miRNAs and 2 down-regulated miRNAs during adipocytic differentiation. Skårn et al. [17] found 66 differentially expressed miRNAs in adipogenesis. Ortega et al. [18] isolated fat cells from both lean (BMI<25.0Kg/m^2^, n=3) and obese (BMI>30.0Kg/m^2^, n=3) subjects for miRNA array, and identified 5up-regulated miRNAs and 7 down-regulated miRNAs between lean and obese subjects. Mikkelsen et al. [19] got the data from hMSCs-Ad adipogenesis, which used the same cell line in our study, and detected 17 up-regulated miRNAs and 19 down-regulated miRNAs in mature adipocytes compared to hMSCs-Ad.

Our study found 79 miRNAs (45 up-regulated miRNAs and 34 down-regulated miRNAs) were significantly up- or down-regulated in mature adipocytes compared to SVCs and hMSCs-Ad (Figure 1, Supplementary Table 1). We detected 42 meta-signature miRNAs from these miRNA array data, which were differently expressed miRNAs in mature adipocytes compared to SVCs or MSC (Table 3). According to the sources of cells, we detected the meta-signature miRNAs from human adipocytes and MSC, respectively. We also identified 17 meta-signature differently expressed miRNAs in obese and lean subjects (Table 4).

**Table 3:** Adipocyte differentiation related meta-signature miRNAs

miRNA name	Our study typeD15 Vs D0	other studies type	Reference ID
hsa-let-7a	UP	UP	[17]
hsa-let-7c	UP	UP	[17]
hsa-let-7d	UP	UP	[17]
hsa-let-7e	UP	UP	[17]
hsa-let-7f	UP	UP	[17]
hsa-miR-10b	UP	UP	[12] [16]
hsa-miR-1201	UP	UP	[15]
hsa-miR-132	UP	UP	[12]
hsa-miR-137	UP	UP	[19]
hsa-miR-152	UP	UP	[16]
hsa-miR-154	UP	UP	[15]
hsa-miR-15a	UP	UP	[16]
hsa-miR-181a	UP	UP	[12),[17]
hsa-miR-193b*	UP	UP	[16]
hsa-miR-26a	UP	UP	[16), [17]
hsa-miR-26b	UP	UP	[16]
hsa-miR-30a	UP	UP	[16),[19]
hsa-miR-30a*	UP	UP	[19]
hsa-miR-30b	UP	UP	[16]
hsa-miR-30c	UP	UP	[16]
hsa-miR-30d	UP	UP	[12] [15],[16],[19]
hsa-miR-365	UP	UP	[16]
hsa-miR-374a	UP	UP	[16]
hsa-miR-374b	UP	UP	[16]
hsa-miR-378	UP	UP	[19]
hsa-miR-378*	UP	UP	[19]
hsa-miR-498	UP	UP	[12), [13]
hsa-miR-642	UP	UP	[12), [19]
hsa-miR-663	UP	UP	[12]
hsa-miR-18a	DOWN	DOWN	[12]
hsa-miR-27a	DOWN	DOWN	[17]
hsa-miR-92a	DOWN	DOWN	[17]
hsa-miR-106b	DOWN	DOWN	[17]
hsa-miR-130b	DOWN	DOWN	[17]
hsa-miR-155	DOWN	DOWN	[13]
hsa-miR-221	DOWN	DOWN	[17]
hsa-miR-222	DOWN	DOWN	[13]
hsa-miR-218-2*	DOWN	DOWN	[13]
hsa-miR-494	DOWN	DOWN	[15]
hsa-miR-629*	DOWN	DOWN	[13]
hsa-miR-758	DOWN	DOWN	[13]
hsa-miR-1825	DOWN	DOWN	[21]

**Table 4:** Obese related meta-signature miRNAs

miRNA name	type D15 Vs D0	obese vs normal	Reference ID
hsa-miR-92a	DOWN	DOWN	[14]
hsa-miR-758	DOWN	DOWN	[13]
hsa-miR-663	UP	UP	[12]
hsa-miR-642	UP	UP	[12]
hsa-miR-629*	DOWN	DOWN	[13]
hsa-miR-498	UP	UP	[12], [13]
hsa-miR-484	DOWN	DOWN	[18]
hsa-miR-30d	UP	UP	[12]
hsa-miR-218-2*	DOWN	DOWN	[13]
hsa-miR-199a-5p	UP	UP	[18]
hsa-miR-185	DOWN	DOWN	[18]
hsa-miR-181a	UP	UP	[12]
hsa-miR-132	UP	UP	[12]
hsa-miR-130b	DOWN	DOWN	[18]
hsa-miR-10b	UP	UP	[12]
hsa-miR-106b	DOWN	DOWN	[12]
hsa-let-7i	DOWN	DOWN	[14]

### GO and pathway analysis

**Figure 4:**
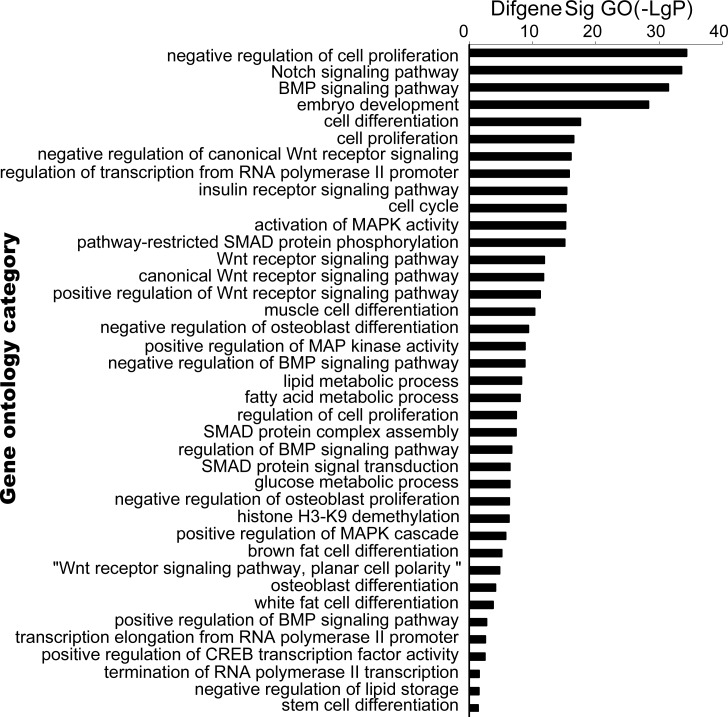
Bioinformatic analysis target genes of meta-signature miRNAs GeneCodis web tool was used to perform statistical analysis of over represented GO terms to predict target genes of meta-signature miRNAs. Cellular processes were sorted by score (−log [*P* value]). The highly positive score set included genes involved in cell differentiation, cell cycle, cell proliferation and Wnt signaling pathway of GO terms.

**Figure 5:**
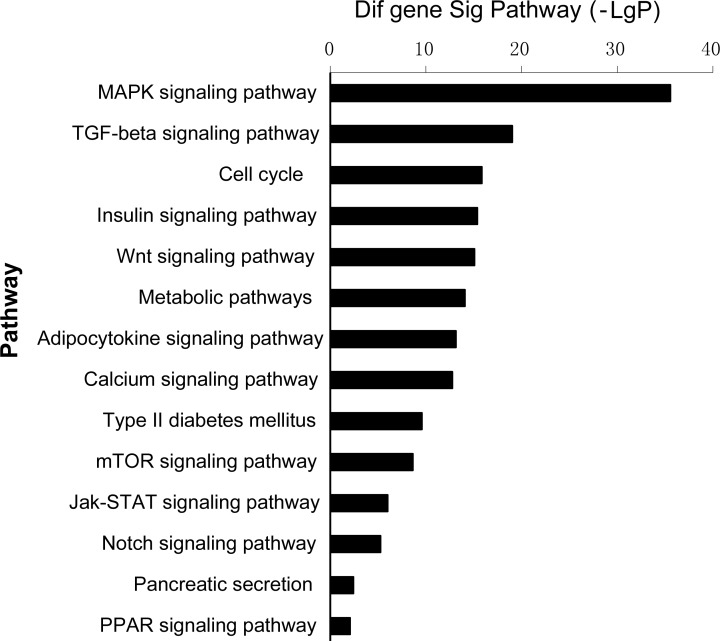
Pathway analyses for predicting target genes of meta-signature miRNAsin Kyoto Encyclopedia of Genes and Genomes (KEGG) database Almost all of the meta-signature miRNAs were particularly involved in Wnt signaling, MAPK signaling, mTOR signaling and ECM-receptor interaction.

To determine which GO and pathways of meta-signature miRNAs are involved in adipogenesis, the predicted gene targets were compared against pathways in the Go and Kyoto Encyclopedia of Genes and Genomes (KEGG) database. The predicted targets were found to be significantly over represented in cell differentiation, cell cycle, cell proliferation and Wnt signaling pathway of GO terms (Figure 4, Supplementary Table 3). The KEGG enrichment pathway analysis of our microarray data revealed that almost all of the meta-signature miRNAs were particularly involved in Wnt signaling, MAPK signaling, mTOR signaling and ECM-receptor interaction. Several genes in the Wnt signaling set (WNT4; LRP6; DAAM2; FRAT2;SKP1; PRKX; FZD3; FZD9; NFATC1; NFAT5; CHP; PPP3CA) were also targeted by multiple miRNAs as part of a complex regulatory network (Figure 5, Supplementary Table 4).

### miRNA-GO-NetWork

**Figure 6:**
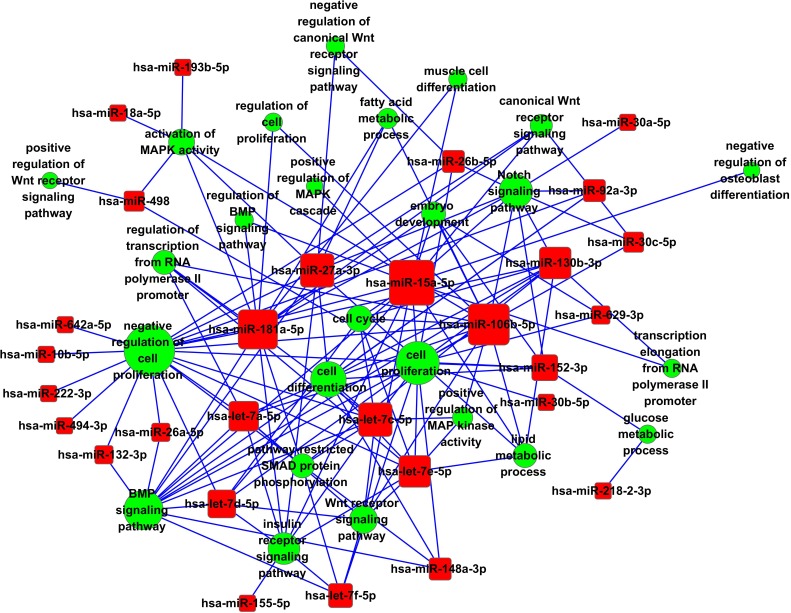
GO-NETwork miRNA-target gene network analysis Red box nodes represented miRNAs and green cycle nodes represented target genes. Edges indicated the inhibitive effect of miRNAs on target genes.

To identify which miRNAs play a critical role in adipogenesis, miRNA-Go-NetWork was analyzed by bioinformatics analysis. As shown in Fig 6, hsa-miR-15a-5p, hsa-miR-106b-5p, hsa-miR-181a-5p, hsa-let-7 family, hsa-miR-27a-3p, hsa-miR-130b -3p, hsa-miR-152/148a-3p and hsa-miR-26b-5p got the highest degree means, which indicated that these miRNAs had more weight in adipogenesis than others.

## DISCUSSION

In this study, we reported the differential miRNA expression in mature adipocyte compared with SVCs or hMSCs-Ad and most of differentially expressed miRNAs located in obesity related chromosomal regions. The expression pattern of differential miRNAs between SVCs Vs. mature adipocyte and hMSCs-Ad Vs. mature adipocyte are similar, indicating that these differential miRNAs were indeed involved in adipogenesis. Our results are in line with the study from Alajez et.al. [16], describing 36 up-regulated miRNAs and 2 down-regulated miRNAs in mature adipocytes compared to hMSCs-TERT.

miR-146b-5p, miR-335, miR-424, miR-1275, miR-155, miR-1268, miR-148a, miR-26b, miR-132, miR-365, miR-1908, miR-93 and miR-720 were identified more than 3-fold changes in mature adipocyte compared with SVCs or hMSCs-Ad. More importantly, the highest fold change was observed in miR-146b in mature adipocyte. Ahn et al. reported that miR-146b identified as a positive modulator of adipocyte differentiation via suppression of sirtuin 1 (SIRT1) [20]. Interestingly, our previous study reported that miR-146b is highly expressed in the adipose tissue of obese mouse models such as DIO, diet-induced obesity, ob/ob, and db/db. This finding has implicated miR-146b as a positive regulator of accelerated adipocyte differentiation through modulation of SIRT1 and KLF7 [6]. Indeed, our previous study found that miR-148a promotes hMSCs-Ad to differentiate to mature adipocyte by targeting Wnt1 [5]. Additionally, our group [7] has also suggested that inhibited miR-26b inhibits adipogenic differentiation in human preadipocytes, and miR-335, as a potential adipogenic miRNA, is involved in adipose tissue inflammation and is highly expressed in mature 3T3-L1 [21]. Therefore, our present and previous studies bring in valuable information with respect to human obesity pathology because we have demonstrated that miR-148a, miR-146b, miR-26b and miR-335 are dysregulated in the process of adipocyte differentiation.

Since obesity-related hyperplasia and hypertrophy are often associated with various metabolic disorders, the detail understandings of the molecular events regulating adipogenesis and lipid metabolism are important. Ortega et al. [18] isolated fat cells from both lean (BMI<25.0Kg/m^2^, n=3) and obese (BMI>30.0Kg/m^2^, n=3) subjects for miRNA array and then differentiated to mature adipocyte, and identified 5 up-regulated miRNAs and 7 down-regulated miRNAs between lean and obese subjects. In addition, 65% (51/79) differentially expressed miRNAs from our data located in obesity related chromosomal regions which was published in 2005 (11), indicating that these differentially expressed miRNAs have a close relationship with obesity.

Although, miRNAs play an important role in the progression of obesity and adipogenesis, different microarray techniques, technical approaches, tissue or cellular models, and small sample size led to inconsistent findings in previous studies. Thus, the present observation reported that 42 differentially expressed miRNAs (meta-signature miRNAs) in mature adipocytes compared to SVCs or hMSCs-Ad. The number of meta-signature miRNAs studies reported varied greatly but at least 12 deregulated miRNAs were reported in each study. Ortega et al. [18] identified that miR-484 and miR-130b were lowly expressed in human subcutaneous adipose from obese and type 2 diabetes mellitus (DM), miR-199a-5p, miR-221, miR-125b and miR-1229 were lowly expressed, indicating that these miRNAs play a key role in obesity. However, this study identified miR-143 was down regulated in mature adipocyte, which conflicted with other studies [22]. Furthermore, this microarray data was not verified by qRT-PCR, thus, this result may be influenced by experimental error, individual difference and species variation.

The KEGG enrichment pathway analysis of our microarray data revealed that almost all of the meta-signature miRNAs were particularly involved in Wnt signaling, MAPK signaling, mTOR signaling and ECM-receptor interaction. Wnt signaling was first recognized as a possible negative regulator of adipogenesis when Wnt1 expression decreased significantly during adipocyte differentiation progress [23]. In particular, our study found that miR-148a through regulating Wnt1, but not Wnt10b, promotes hMSCs-Ad differentiation [5]. ERK, p38 and JNK signaling pathway belong to MAPK signaling, which are intracellular signaling pathways that play a pivotal role in many essential cellular processes such as proliferation and adipocyte differentiation [24]. Importantly, Esau et al. [25] reported that miR-143 promoted adipocyte differentiation by inhibiting ERK5. mTOR signaling belongs to the phosphoinositide 3-kinase (PI3K)-related kinase family, and promotes adipogenesis in white adipocytes [26]. Previous study reported that miR-17-92 cluster has been reported to be upregulated during the clonal expansion stage of adipocyte differentiation, posi­tively regulating adipogenesis by targeting the tumor suppressor RB2/p130 [27] and knockdown of miR-106b and miR-93 significantly induced the expression of brown fat-specific genes and promoted the accumulation of lipid-droplet in differentiating brown adipocytes. In addition, ectopic expression of miR-106b and miR-93 suppressed the mRNA level of Ucp1 [28]. miR-93 is a highly expressed PCOS patients' adipocyte tissue and downregulates GLUT4 gene expression, suggesting that it plays a role in the IR of PCOS [15]. Interestingly, let-7-mediated repression of adipogenesis was also found to promote osteogenesis [29]. In the present study, we found that hsa-miR-15a-5p, hsa-miR-106b-5p, hsa-miR-181a-5p, hsa-let-7 family, hsa-miR-27a-3p, hsa-miR-130b-3p, hsa-miR-152/148a-3p and hsa-miR-26b-5p got high degree means, which indicated that these miRNAs had a great weight in adipogenesis than others.

We concluded that meta-signature miRNAs involved in adipocyte differentiation and provided pathophysiological roles and novel insight into obesity and its related metabolic diseases.

## MATERIALS AND METHODS

### Cell culture and adipocyte differentiation

Human stromal vascular cells (SVCs, also called “primary” preadipocytes, Cat.No.7210, ScienCell Research Laboratories, San Diego, CA) and Human adipose-derived mesenchymal stem cells (hMSCs-Ad; Cat.No.7510, ScienCell Research Laboratories, San Diego, CA) were maintained in Preadipocyte Medium (PAM) (Cat.No.7211, ScienCell Research Laboratories) and Mesenchymal Stem Cell Medium (MSCM) (Cat.No.7501, ScienCell Research Laboratories) supplemented with 5% fetal bovine serum, 1% mesenchymal stem cell growth supplement (Cat. No. 7501) and 1% penicillin/streptomycin solution at 37°C in a humidified atmosphere under 5% CO2. To induce differentiation, HMSC-Ad cells were incubated in serum-free MSCM supplemented with 50 nM insulin, 100 nM dexamethasone, 0.5 mM 3-isobutyl-1-methylxanthine, and 100 μM rosiglitazone (Day 0) and the medium was replaced every 2 days over 4 days. Thereafter, cells were incubated in serum-free MSCM supplemented with 50 nM insulin and replaced every 2 days until lipid accumulated in cells.

### miRNA microarray

RNA was labeled and hybridized to μParaflo® human miRNA microarrays following the manufacturer's instructions by LC Sciences (MRA-1001, miRHuman_14). Each chip contains 894 specific microRNA probes (repeated four times for each probe) and 50 controlled probes (according to the purpose of experiment, each probe is repeated 4-16 times). miRNA microarray analysis was performed using RNA samples from human preadipocyte, human adipose tissue-derived mesenchymal stem cells (hMSCs-Ad) and mature adipocyte.

### RNA isolation and analysis of miRNAs expression by RT-quantitative PCR (qPCR)

Total RNA was prepared from SVCs or hMSCs-Ad at varying intervals after induction of adipocyte differentiation using TRIzol (Invitrogen, Carlsbad, CA) according to the manufacturer's protocol, followed by DNase treatment (TaKaRa, Japan). The quality and concentration of RNA was assessed by Nanodrop2.0 (Thermo Fisher Scientific, Waltham, MA). 240 ng of total RNA was reverse transcribed using the high capacity reverse transcription kit (Applied Biosystems, Life Technologies) according to the manufacturer's protocol. One microliter of cDNA was then amplified by real-time qPCR using the Taqman universal PCR master mix (Applied Biosystems) and specific primers in an Applied 7500 QPCR System (Applied Biosystems). miRNA expression levels were normalized using the snoU6 miRNA expression level as internal controls. Each sample was measured in triplicate, and the gene expression levels were calculated using the 2^−ΔΔct^ method.

### Study selection and data extraction

A systematic literature search was performed for the identification of adipogenic or obesity miRNA expression profiling studies using a two-level search strategy. First, we undertook a web-based search in Gene Expression Omnibus (GEO, http://www.ncbi.nlm.nih.gov/geo/) using search term (“adipocyte OR adipogenesis OR fat cell OR adipocyte differentiation OR obesity”[Mesh]) AND (“MicroRNAs”[Mesh]) AND “Humans”[Mesh]. To perform a comprehensive retrieval, searching in ArrayExpress (www.ebi.ac.uk/arrayexpress), Pubmed and Embase database were also performed. Second, the reference lists of all relevant and existing studies were reviewed through a manual search for further identification of potential relevant studies.

Abstracts were screened carefully and full texts of relevant potential abstracts were evaluated. Studies with original experimental design that analyzed the miRNA expression profiling in human between adipose tissues or human adipocytes were included. Meanwhile, studies were not eligible for meta-analysis if they met the following selection criteria: 1) using human preadipocytes or SVCs or hMSCs-Ad or hMSC cell, 2) preselected candidate genes research, 3) differentiating to mature adipocyte.

The lists of statistically significant expressed miRNAs were extracted from publications. Authors were contacted when the lists could not be obtained. All miRNA names were standardized through miRBase version 20.0. miRNAs that cannot be related to either −3p or −5p in miRBase were designated with hsa-miR-*, such as hsa-miR-210. Those identified as dead entry in miRBase retained their names mentioned in the literatures.

### Integrative identification of miRNA targets

The meta-signature miRNAs were selected for target prediction by using TargetScan database version 6.2 [30] and miRanda (http://www.microrna.org) were used to predict the target consensus sequences for meta-miRNA.

### Enrichment analysis

To identify the pathways of predicted miRNA targets, Kyoto Encyclopedia of Genes and Genomes (KEGG), Panther pathways and Gene Ontology terms were carried out with GeneCodis web tool (http://genecodis.dacya.ucm.es/) [31]. Use the KEGG database to build the network of genes in accordance with the relationship among the genes, proteins and compounds in the database. GO analysis was applied to analyze the main function of the target genes of differential expression miRNA according to the Gene Ontology which is the key functional classification of NCBI [32]. Enrichment provides a measure of the significance of the function: as the enrichment increases, the corresponding function is more specific, which helps us to find those GOs with more concrete function description in adipogenesis.

### miRNA-GO network

MiRNA-GO network is built according to the relationship of miRNAs and significant GOs. In the miRNA-GO network, the circle represents GO and the shape of red square represents miRNAs, and their relationship is represented by one edge. The center of the network is represented by degree. Degree of miRNA means the number of links one miRNA regulates GOs. The core miRNAs in the network always have the highest degrees.

### Statistical analysis

The lists of miRNAs were extracted based on statistical test *P*-values (<0.05 was considered significant). The representatives of replicate experiments were shown in the figures and the data were shown as the means ± SEM. The statistical analyses were performed using independent Student's t-tests. In case of false positive results, Bonferroni correction was performed.

## SUPPLEMENTARY MATERIAL








